# The risks of physicians’ conformism: reflections from the opioid overflow

**DOI:** 10.1186/s13052-021-00967-z

**Published:** 2021-01-15

**Authors:** Luisa Cortellazzo Wiel, Giorgio Cozzi, Egidio Barbi

**Affiliations:** 1 University of Trieste, Trieste, Italy; 2 Institute for Maternal and Child Health – IRCCS “Burlo Garofolo”, Trieste, Italy

**Keywords:** Opioids, Addiction, Pain measurement, Acute pain, Chronic pain

## Abstract

Opioid-related mortality in adolescents is spreading in the US, with prescription opioids playing a crucial role in the development of addiction. We traced back to the process leading to the so called “opioid overflow”, trying to identify any modifiable attitude.

Since the late 1990s, pain was labelled as the “fifth vital sign” and its proper management was prompted, encouraging the use of opioids for any pain scored at a Numerical Rating Scale (NRS) of 7 or higher. This assumption has some remarkable limitations. NRS is a proxy of pain severity in children, and pain measurement should be strengthened by a more comprehensive pain evaluation. Moreover, while remaining a fundamental therapeutic right of patients suffering postoperative or chronic severe pain, opioids show no evidence of superiority respect to non-opioid regimens in the management of pain from several acute conditions.

Italy, as other European countries, is often reluctant to the use of opioids, even when highly recommendable, missing the opportunity of properly treating those selected patients with severe pain. Both attitudes can be viewed as the result of an extreme simplification of the complex process of pain evaluation and treatment, by means of a ‘one-size-fits-all’ approach.

This highlights the need for a systematic and patient-tailored attitude to children in pain, avoiding applying guidelines without question. Good clinical practice must rely on guidelines, which, however, as often based on partial and insufficient data, can be questioned by emerging new evidence, and should not substitute our rational thinking, and capability to understand each patient, avoiding excessive conformism.

## Text

Opioid-related fatalities in adolescents aged 15 to 24 in the US have exceeded those from tumours, cardiopathies and congenital malformations combined, reaching an overall figure of 8.6/100.000 per year, and representing one of the leading causes of death in the young [1]. The vast majority of these deaths are estimated to be somehow related to self- or familial-prescribed opioids, which, if leftover and not properly disposed of, act as a reservoir or gateway for non-prescription use [2]. In the US, up to 45% of adolescents have received at least one opioid prescription by the age of 18 [3], and among heroin users, 80% report having been exposed to opioid pain medications before the development of addiction [4]. Accordingly, a recent Swedish study demonstrated a 30% relative greater risk of substance-related morbidity in opioid medical treatment recipients compared to non-steroidal anti-inflammatory drugs (NSAID) recipients [5]. Overall, in an Emergency Department (ED) perspective, children’s and adolescents’ prescriptions have increased as much as 30% from 2001 to 2010 in the US, partly contributing to the spread of the opioid epidemic [6]. However, the number of outpatient opioid prescriptions in the Paediatric Emergency Departments is limited and significantly lower than that observed in children admitted to the General Emergency Departments [7].

In Italy, an increase in opioid overdose-related deaths has been observed from 1984 to 2000, with one peak in 1991, and a further one in 1995–1996, followed by a progressive decline; however, this rise has been mostly limited to older subjects, aged 25 to 44 years, while among younger users, aged 15 to 24, the incidence has shown a clear reduction after the 1991 peak, until the figure of 0.07/100.000 per year in 2017 (http://dati.istat.it/Index.aspx?DataSetCode=DCIS_CMORTE1_EV#). According to a retrospective study, involving 18 Italian EDs between October 2014 and January 2015, only 14% of 1417 of children received opioids for the management of acute non-procedural pain, with codeine plus acetaminophen being the most frequently prescribed drug (52%), followed by morphine (27%) and tramadol (21%) [8].

Opioid overflow can be understood as a paradigm shift considering that, less than 2 years ago, we were dealing with the emerging notion of the large under-recognition and under-treatment of pain, especially in children, and its detrimental neurobiological and psychosocial long-term effects. Since the late 1990s, pain has been labelled as the “fifth vital sign”, and its proper recognition and management have become quality indicators in healthcare. In this framework, a large amount of literature has been produced, on both pain assessment and treatment in the paediatric population: several self- and caregiver-reported numerical scales for pain measurement have been developed, and the use of opioids has spread for the management of severe pain. Since then, we assumed that many kinds of pain scored at a Numerical Rating Scale (NRS) of 7 or higher in a broad range of patients deserved the administration of opioids for optimal management. This assumption has some remarkable limitations.

The first one rises from the modality of detection of pain itself. NRS is a proxy of pain severity in children, and as such needs to be interpreted within the context. Although the subject that is suffering is the only one endorsed with the right to express the amount of pain they are perceiving, the experience of pain results from both the peripheral nociceptive stimulus and its central elaboration: the latter depends upon a variable balance between pain-enhancing factors, including fear, anxiety and memory of previous pain experiences, and protecting elements, like the intellective level, resilience and sense of belonging.

Thus, to obtain an accurate esteem of pain severity in children, pain measurement should be strengthened by a more comprehensive pain evaluation and treatment planning, distinguishing between acute and long-term treatments.

The issue at stake is that the routine use of opioids in the management of pain from the vast majority of acute paediatric conditions, both in the ED and after discharge, is not supported by any evidence of their superiority respect to non-opioid regimens. In fact, the most frequent paediatric conditions associated with severe pain largely benefit from non-opioid treatments, namely NSAIDs or even acetaminophen at appropriate dosages. Some examples are musculoskeletal pain from traumas or fractures [9], renal colic, migraine attacks, and acute abdominal pain [10]. On the other hand, physicians are also increasingly dealing with children reporting severe pain, which rises from somatic conditions, which will thus not benefit from pharmacological treatments [11]. If inappropriately treated, these patients could even be at higher risk of developing a substance use disorder due to the frequent comorbidity with anxiety and depression, and to their risk of isolation from peers.

The World Health Organization (WHO) analgesic ladder, advocating the optimization of non-opioid pain medications before adding opioids, can be relied upon to guide the management of pain in children [12]. Accordingly, opioids represent a compelling opportunity for the control of severe pain, in selected emergency situations (e.g intranasal fentanyl in children with burns or misaligned fractures attending the ED) and acute/subacute, especially post-operative, settings.

On the other hand, opioids remain a fundamental therapeutic right of patients suffering any chronic severe pain deriving from different long-term of life-long conditions (e.g. cancer, progressive, and degenerative disorders) either as a first-line or after the failure of first-line alternative treatments. These patients with chronic severe pain force us to confront with another consideration on NRSs: when we ask children to score their level of pain from none to “the worst pain you can imagine”, we should be aware that the upper limit of pain imaginable by any child depends on their previous pain experiences, and this unavoidably influences their NRS reported score.

We could interpret the easiness of opioids prescription, and at least a part of the opioid epidemic, as the result of an extreme simplification of the complex process of pain evaluation and treatment, by means of a ‘one-size-fits-all’ approach. On the other hand, Italy, as other European countries, has been (and often remains) reluctant to implement the use of opioids, even when highly recommendable. This attitude, as the opposite one, can be viewed as the result of a conformist approach to the management of pain. Remarkably, in this perspective, Italian physicians’ approach causes the loss of opportunity of properly treating those selected patients with severe pain.

Throughout history, conformist attitudes have resulted from a variable combination of a need for standardization and a difficulty in abandoning previously established approaches. Although practice standardization aims to improve patients’ outcome, the possible drawback of a lack of a personalized approach to each patient, is also well known [13]. Accordingly, while remaining a crucial instrument for good clinical practice, guidelines are often based on partial and insufficient data, questionable by emerging new evidence, and thus should not be applied without question. Moreover they should not be confused with non-evidence-based “medical fashions”, habits and dogmas, moulded by tradition, and difficult to be questioned despite proof of their ineffectiveness [14]. Indeed, we must accept that the progress of medicine is built from steps forward, which unavoidably challenge our previous attitudes. Not without reason, evidence-based medicine is defined as the judicious use of the current best evidence in clinical practice, by integrating scientific findings with the individual expertise, made up of a variable combination of personal experience, reasoning and peer discussion [15].

We suggest that, regardless of our working setting, we should address pain in children through a systematic and patient-tailored approach. The acritical implementation of both NRSs, and treatment guidelines, should not substitute our rational thinking, and capability to understand each patient. Physicians should be aware of the risks of an excess of conformism.

## Data Availability

N/A

## Abbreviations

ED: Emergency Department
NSAID: Non-steroidal anti-inflammatory drugs
NRS: Numerical Rating Scale
